# The High Radiosensitizing Efficiency of a Trace of Gadolinium-Based Nanoparticles in Tumors

**DOI:** 10.1038/srep29678

**Published:** 2016-07-14

**Authors:** Sandrine Dufort, Géraldine Le Duc, Murielle Salomé, Valerie Bentivegna, Lucie Sancey, Elke Bräuer-Krisch, Herwig Requardt, François Lux, Jean-Luc Coll, Pascal Perriat, Stéphane Roux, Olivier Tillement

**Affiliations:** 1 Thérapie ciblée, Diagnostic précoce et Imagerie du cancer, INSERM/UJF U823, Institut Albert Bonniot, 38706 La Tronche Cedex, France; 2 Nano-H S.A.S, 2 Place de l’Europe, 38070 Saint Quentin-Fallavier, France; 3 ID17 Biomedical Beamline and ID21 Beamline, European Synchrotron Radiation Facility, 38000 Grenoble, France; 4 Institut Lumière Matière, UMR 5306 CNRS-UCBL, Université Claude Bernard Lyon 1, 69622 Villeurbanne Cedex, France; 5 Matériaux Ingénierie et Science, UMR 5510 CNRS−INSA, INSA de Lyon, 69621 Villeurbanne Cedex, France; 6 Institut UTINAM, UMR 6213 CNRS-UFC, Université de Franche-Comté, 25030 Besançon Cedex, France

## Abstract

We recently developed the synthesis of ultrasmall gadolinium-based nanoparticles (GBN), (hydrodynamic diameter <5 nm) characterized by a safe behavior after intravenous injection (renal clearance, preferential accumulation in tumors). Owing to the presence of gadolinium ions, GBN can be used as contrast agents for magnetic resonance imaging (MRI) and as radiosensitizers. The attempt to determine the most opportune delay between the intravenous injection of GBN and the irradiation showed that a very low content of radiosensitizing nanoparticles in the tumor area is sufficient (0.1 μg/g of particles, *i.e.* 15 ppb of gadolinium) for an important increase of the therapeutic effect of irradiation. Such a promising and unexpected result is assigned to a suited distribution of GBN within the tumor, as revealed by the X-ray fluorescence (XRF) maps.

Owing to their large range of properties which can be accurately tuned by the chemical composition, the shape and the dimensions, multifunctional nanoparticles appear as promising candidates for image-guided therapy since they can be designed for combining various imaging modalities and remotely controlled therapeutic activity^1^,^2^,^3^,^4^,^5^. The data collected by medical imaging about the biodistribution of the nanoparticles can therefore be useful for initiating the activation of the therapeutic effect. From the intense research activities devoted to the development of multifunctional nanoparticles for cancer therapy, some general trends can be determined for a significant increase in life span (ILS). Whatever the therapeutic strategy implemented for the eradication of a solid tumor, the most essential rule rests on the preservation of the surrounding healthy tissue^6^,^7^,^8^,^9^. This implies that the therapeutic effect occurs only in the tumor. The accumulation of therapeutic agents in tumor can be preferentially achieved after intravenous injection by enhanced permeability and retention (EPR) effect (passive targeting, owing to the great porosity of the neo-vessels which irrigate the solid tumors) or by an active targeting which also rests on the EPR effect but requires the functionalization with biotargeting groups^10^,^11^. In contrast to the molecules, the presence of the nanoparticles in the healthy tissue is generally limited to the vascular network because the nanoparticles are sufficiently large for avoiding their extravasation from normal blood vessels and therefore their diffusion outside the bloodstream in the healthy tissue^6^,^7^,^8^,^9^,^10^,^11^. However, the circulation of the nanoparticles in the vessels irrigating the healthy tissue must be taken into account in order to preserve healthy tissue during a treatment. The influence of the therapeutic agents content in the surrounding healthy tissues on the survival was highlighted by a previous study performed on 9L gliosarcoma (9LGS) bearing rats treated by microbeam radiation therapy (MRT) after intravenous injection of gadolinium-based nanoparticles (GBN)^12^. These nanoparticles whose hydrodynamic diameter is less than 5 nm have been designed for a complete removal from the body by renal clearance. They are obtained from polysiloxane encapsulated gadolinium oxide nanoparticles. The functionalization of the thin polysiloxane shell by linear (DTPA derivative) (DTPA: 2-[Bis[2-[bis(carboxymethyl)amino]ethyl]amino]acetic acid (also called: diethylenetriaminepentaacetic acid) or macrocyclic (DOTA derivative) (DOTA: 1,4,7,10-tetraazacyclododecane-1,4,7,10-tetraacetic acid) polyaminocarboxylate ligands induces the dissolution of the gadolinium oxide core. As a result, the colloidal suspension is composed of nanosized polysiloxane platforms functionalized by gadolinium chelates since the released gadolinium ions are captured by the ligands^13^,^14^. The presence of gadolinium confers to GBN a behavior of positive contrast agents for magnetic resonance imaging (MRI) and of radiosensitizers^12^,^13^,^14^,^15^,^16^,^17^,^18^,^19^,^20^,^21^. The main conclusions of this study were that (i) the follow up of GBN by MRI is very useful for guiding the radiotherapy and (ii) the radiosensitizing effect of GBN improves the treatment by MRT only if the GBN content is both sufficiently high in the tumor and sufficiently low in the surrounding healthy tissue^12^. Since previous biodistribution studies demonstrated that GBN are confined in the blood vessels which irrigate the healthy tissue and are removed from body through renal clearance (no extravasation and hence no undesirable accumulation in healthy tissues are observed), their presence in the healthy tissue is fortunately more transient than in the tumor^13^,^14^. As a result, there is a delay between the injection and the irradiation from which the radiosensitization improves the radiotherapy. In the case of GBN, an important ILS was observed when the irradiation was performed 20 minutes after intravenous injection^12^. Since the potential of GBN for image-guided radiotherapy was confirmed, one of our goals consists in the determination of the optimal delay when deleterious effect on the healthy tissues is insignificant (*i.e.* at least 20 minutes after the injection). This should also correspond to a better distribution since a longer delay between the injection and the irradiation should lead to a greater penetration of particles in the tumor. Our attempt to plentifully exploit the promising potential of GBN for image-guided radiotherapy provides interesting, unexpected and useful information (collected by MRI, ICP analysis and XRF mapping) for improving the survival time of the tumor-bearing animals.

## Results

**Figure 1:**
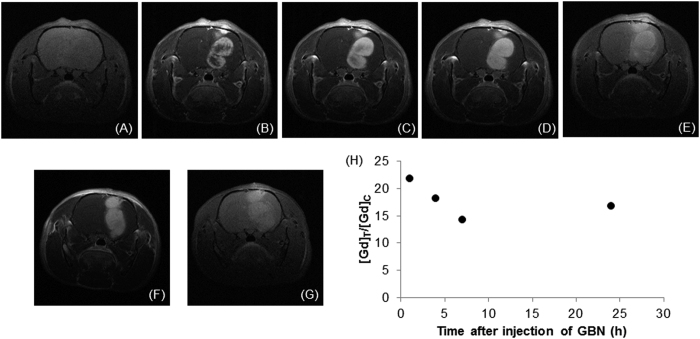
T_1_-weighted images of the brain of two 9LGS-bearing rats. The images were acquired (**a**) before and (**b**) 1 min, (**c**) 10 min, (**d**) 15 min and (**e**) 1 h after intravenous injection of GBNs for rat 1, (**f**) 15 min and (**g**) 24 h after intravenous injection of GBNs for rat 2 (56 μmol Gd). (**h**) Temporal evolution of gadolinium concentration ratio between tumor and contralateral region (T/C ratio).

Owing to the presence of gadolinium ions, the distribution of GBN within the tumor can be monitored by MRI^17^,^18^,^19^,^20^,^21^. The tumor is not visible on T_1_-weighted images acquired before the intravenous injection of GBN whereas a positive contrast enhancement of the outer part of the tumor is observed 1 min after the injection (Fig. 1a,b).

**Figure 2:**
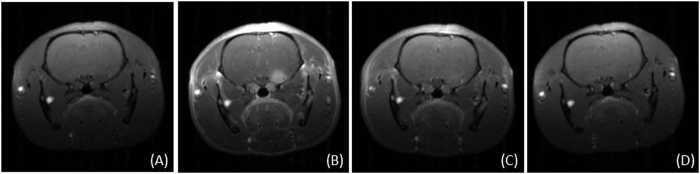
T_1_-weighted images of the brain of 9LGS-bearing rats (**a**) before and (**b**) 20 min, (**c**) 1 h and (**d**) 24 h after intravenous injection of Dotarem^®^ (56 μmol Gd).

The delineation of the tumor is therefore quickly achieved. Moreover the examination of the images acquired during the first 15 min clearly shows the extension of the bright zones within the tumor, from the outer part toward the center of the tumor (Fig. 1b–d,f). These MR images reveal the gradual diffusion of the GBN which induce the positive contrast enhancement within the tumor. After this first step of tumor filling, the images are less and less contrasted (Fig. 1e,g) indicating a decrease in the amount of GBN in the tumor. However the tumor remains entirely visible up to 24 h after the intravenous injection while the positive contrast in the contralateral region remains very low. This indicates that GBN go across blood-brain-barrier (BBB) only when the latter is disrupted (*i.e.* only in the tumor zone)^22^,^23^,^24^. The retention in the tumor for at least 24 h can be assigned to the particulate character of GBN^25^. The comparison between GBN and a molecular contrast agents for MRI (Dotarem^®^, commonly used in clinical examinations) shows indeed that the positive contrast enhancement is visible for a shorter duration in the case of Dotarem^®^ (less than 1h after the intravenous injection of the contrast agents) owing to the rapid renal clearance of molecules (Figs 1 and 2).

The elemental analysis of the whole brain confirms the decrease of GBN content in the brain as a function of time until very low values (from 55 ppb to 1.5 ppb between 1 and 24 h after intravenous injection of GBN). From T_1_-weighted images, the ratio between gadolinium concentration in tumor (T) and in contralateral tissue (C, *i.e.* the healthy part of the brain in left hemisphere) can be determined. The variation of T/C ratio over time reflects the temporal evolution of GBN content in tumor and in healthy tissues in brain. Figure 1h which depicts this variation shows that T/C decreases during the first 7 h after intravenous injection of GBN. This decrease can be assigned to a wash-out process in the tumor. If all nanoparticles were concerned by this phenomenon, the ratio would be equal to zero between 18–19 h after injection (assuming that T/C ratio decreases linearly with time). Since the T/C value at 24 h is significantly different from zero and is similar to the one observed 4–7 h after intravenous injection, the wash-out process should concern only a fraction of GBN. In order to have a clearer idea on the distribution of GBN within the brain and in peculiar within the tumor, post-mortem synchrotron based micro-X-ray fluorescence experiments were performed on brain slices of rats which were sacrificed 1 h and 24 h after intravenous injection of GBN. X-ray fluorescence (XRF) spectra were recorded for each slice within the tumor and within a contralateral region (with a same area).

**Figure 3:**
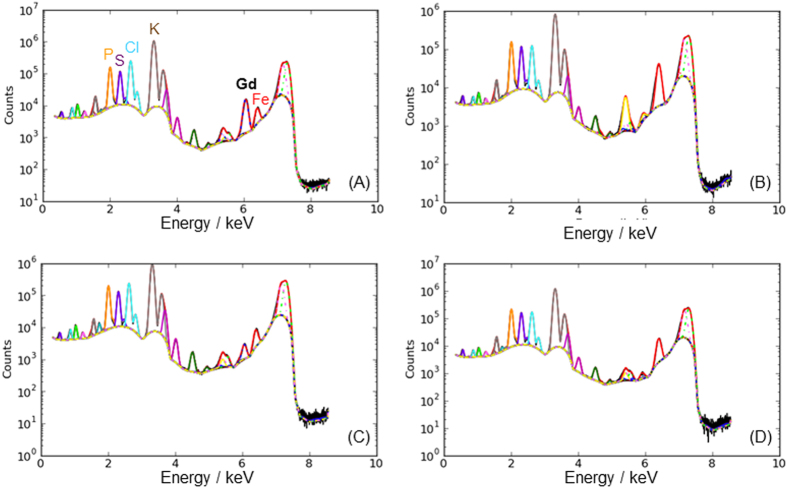
Post-mortem XRF sum spectra of regions of interest in the tumor (**a,c**) and in the contralateral region (**b,d**) from the brain of 9LGS bearing rats sacrificed (**a,b**) 1 h and (**c,d**) 24 h after intravenous injection of GBN.

**Figure 4:**
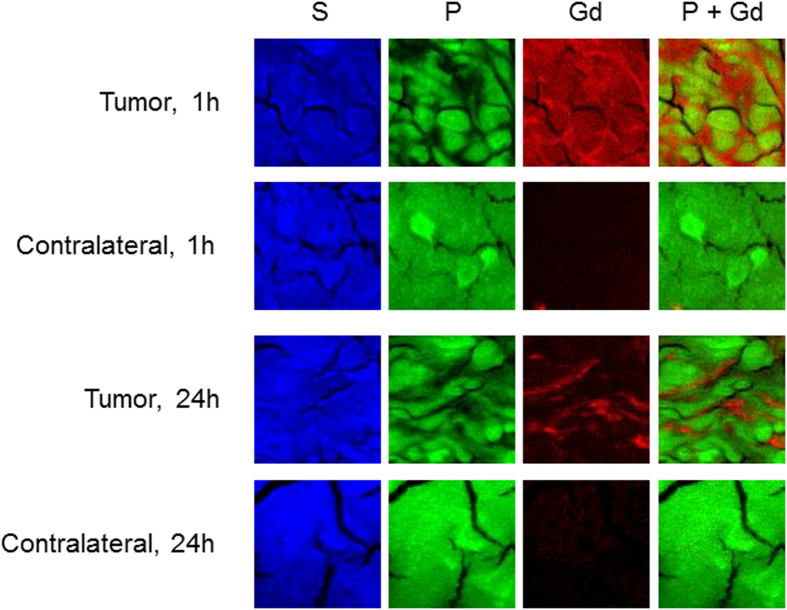
XRF maps of S, P, Gd elements and overlay of Gd and P maps of ROIs in the tumor and the contralateral region from the brain of 9LGS bearing rats sacrificed 1 h and 24 h after intravenous injection of GBN. Field of view 50 × 50 μm^2^, 0.5 μm step size.

Figure 3 displays the resulting XRF sum spectra that exhibit the presence of endogenous elements (phosphorus, sulfur, chlorine, potassium and iron, which XRF K-α emission lines are respectively at 2.0, 2.3, 2.6, 3.3 and 6.4 keV) and the presence of gadolinium (L-α emission line at 6.0 keV) which characterizes the presence of GBN in the brain^26^. Whatever the delay after the injection, the amount of GBN (reflected by the area of the peak at 6.0 keV) is largely more important in the tumor than in the healthy contralateral region. Moreover the content of GBN within the tumor and the contralateral region is lower for the slice extracted from the brain of a rat euthanatized 24 h after injection. These observations which confirm the data retrieved from MR images obviously show the efficient passive targeting of the tumor by GBN (due to EPR effect) and the removal of the particles in course of time. But micro-XRF provides complementary and useful information since a mapping of element can be reconstructed from the spectra recorded for each point of the regions of interest (ROIs). By fitting the XRF emission lines of the different elements in the spectra, a map of the distribution of these elements on a cellular scale can be drawn. For each region of interest, maps for Gd, P and S are displayed on Fig. 4.

Sulfur element indicates the presence of tissue while phosphorus element shows the presence of nucleic acid. It is therefore assumed that the enhanced presence of phosphorus is a fingerprint of the cells in the tissues. The comparison between the maps of P element within the tumor and the contralateral region reveals differences in the distribution of this element. The distribution of phosphorus is more homogenous in the contralateral region (healthy tissue) while this element is concentrated in islets in the tumor. Moreover the contralateral region is characterized by a perfect superimposition between the maps of P and S elements in contrast to the tumor. The mapping of endogenous elements clearly shows the differences in the organization at cellular scale between the tumor and healthy tissue^27^. These differences correlate with the local distribution of GBN: XRF maps show that gadolinium element is not detected in the contralateral region whatever the delay between the administration of GBN and the euthanasia of the rats. In other words, the content in GBN is too low to be visualized in the healthy tissue. On the other hand, gadolinium element is detected in the tumor whatever the delay but the distribution is different for the samples extracted 1 h and 24 h after the intravenous injection of GBN. At 1 h, there is a general background of gadolinium in the tumor with some brighter spots which are located in between the P-rich areas according to the overlay of the P and Gd maps (Fig. 4). When comparing the tumor 24 h and 1 h after the intravenous injection of GBN, a washout effect seems to occur. At 24 h, the background of Gd element has mostly disappeared, but the brighter spots observed around the P-islets at 1 h are still present. We can therefore deduce from these Gd maps and MRI experiments that gadolinium titrated for the whole brain by elemental analysis (1 h and 24 h) is mostly concentrated in the tumor which corresponds to 10% of the total volume of the brain (according to MRI data). The highest gadolinium amount in the tumor can therefore be estimated to only 550 ppb and 15 ppb 1 h and 24 h after intravenous injection of GBN (*i.e.* 5.5 ppm and 0.15 ppm of GBN in the tumor since the weight content of gadolinium in GBN is about 10%).

**Figure 5:**
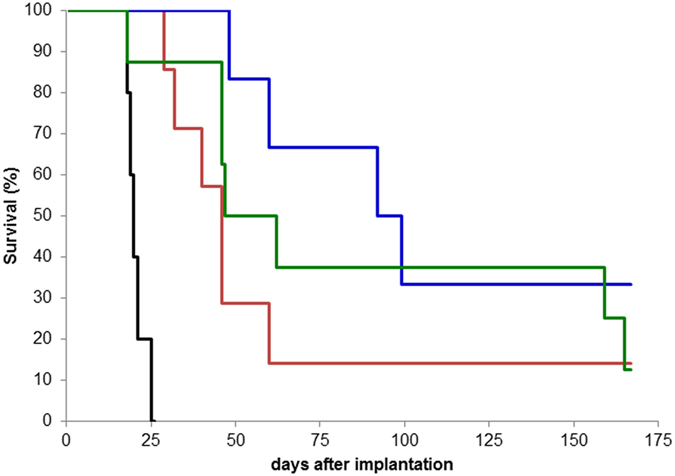
Survival curve comparison obtained on 9LGS bearing rats without treatment (black curve, n = 5 rats), only treated by MRT (red curve, n = 7), and treated by MRT 1 h (green curve, n = 8) and 24 h (blue curve, n = 6) after GBN intravenous injection during 170 days after tumor implantation.

In order to evaluate the radiosensitizing effect of such a low GBN content in the tumor on the survival of diseased animals, the radiotherapy was performed with four randomized groups of 9LGS bearing rats: (i) a control group (the rats are not treated), (ii) a group with only MRT-treated rats, two groups with rats treated by MRT (iii) 1h and (iv) 24 h after intravenous injection of GBN. Owing to the aggressiveness of 9LGS tumor and the sensitivity of the brain, the rats which did not receive any treatment (no irradiation, no administration of nanoparticles) were euthanatized between 18 and 25 days after tumor implantation (median survival time (MeST): 20 days) (Fig. 5). The treatment by MRT leads to an obvious improvement of the survival time since the MeST reaches 46 days (i.e. ILS = 130%)^28^. However MRT is rendered more efficient when GBN are injected 1 h and 24 h prior to the irradiation (MeST: 62 and 95.5 days, ILS: 210 and 377.5% respectively) (Fig. 5). The survival improvement of rats treated by MRT after intravenous injection can be exclusively assigned to the radiosensitizing effect of GBN. Preliminary experiments demonstrated indeed that GBN are not toxic for tumor cells in absence of irradiation. The MeST of 9LGS bearing rats after intravenous injection of GBN (100 μmol Gd (instead of 56 μmol) 10 days after the tumor implantation) without irradiation is the same than the MeST of non-treated rats (26 days).

The longer survival of the diseased rats which were treated by MRT after administration of nanoparticles is assigned to the radiosensitizing effect of GBN which induces an increase of the median survival time (MeST) because GBN are mostly present in the tumor, as revealed by MRI and XRF mapping (Figs 1 and 4)^12^,^29^. In addition to confirm the promising potential of GBN for radiosensitization, this experiment provides important and unexpected information: high ILS is obtained even with very low GBN content (Gd content in the tumor: 15 ppb when MRT was performed 24 h after injection).

## Discussion

The improved survival time observed when the animals are exposed to MRT 24 h after the intravenous injection seems to rest on the peculiar distribution at cellular scale revealed by XRF maps. From the comparison between the maps and the survival curves, we can deduce that a large amount of GBN distributed in the whole tumor is not necessary for increasing the life span of the 9LGS bearing rats. The radiosensitizing effect seems indeed more efficient when the amount is lower but concentrated in P-poor regions. This apparent paradoxical result (higher ILS when GBN content is lower in the tumor) reflects the importance of the location of the nanoparticles in the tumor since the interaction between ionizing radiation and particles should be the same whatever its position. The removal of a large part of nanoparticles from the tumor between 1 and 24 h indicates that the interaction between cancerous cells and these nanoparticles is relatively weak, *i.e.* the particles are not sufficiently close to the cells. As a consequence, the cytotoxic potential of the species produced after absorption of X-ray photons by GBN cannot be plentifully exploited in contrast to the GBN which are still present in the tumor 24 h after the intravenous injection. Their presence after a so long delay might be explained by a stronger interaction between GBN and cancerous cells. In other words, the GBN that are not washed out could be adherent to the surface or even internalized inside the cells^30^. For this reason, the damages caused by the cytotoxic species produced after absorption of X-ray photons by GBN are more important when MRT is performed 24 h after intravenous injection even if the radiosensitizer content is very low (<15 ppb in the tumor 24 h after intravenous injection).

In summary, the effect of MRT can be tremendously enhanced when radiosensitizing GBN are injected before the irradiation. Their content in the tumor is a crucial criterion that must be taken into account for improving the survival time. Nevertheless, a large amount of GBN in the tumor is not necessary for obtaining the best results since the greatest ILS was observed when the content in the tumor is very low but concentrated in P-poor domains of the tumor.

This result is not contradictory with dose responsiveness of radiation based therapy. It only means that a low content of radiosensitizers at the right place can be more efficient than a large amount with a less controlled localization. The washout of a large proportion of particles in the tumor seems to be compensated by a better repartition of the trace of GBNs remaining in the tumor area. The localization of GBN in the tumor is consequently very important in order to ensure a great ILS. If further investigations are needed for determining the mechanisms which allow such a control, this preclinical study, which demonstrates that GBN exerts an efficient radiosensitization at very low content, constitutes however a promising solid basis for envisaging medical application to human patients. Since only a low content of radiosensitizers is necessary for high ILS, this study demonstrates that expensive and time-consuming labeling by biotargeting group is not required when the nanoparticles exhibit both a suited biodistribution and renal clearance.

## Methods

### Preparation of gadolinium-based nanoparticles

Gadolinium-based nanoparticles were synthesized and characterized according to the previously described protocols^12^,^13^,^14^. Further details can be found in Supporting Information.

### Inductively Coupled Plasma-Mass Spectrometry (ICP-MS) analysis

Determination of gadolinium content in brain was performed by ICP-MS analysis (Agilent 7500ce). The determination of gadolinium content in brain required their dissolution in *aqua regia*, prior to heating for 3 hours at 80 °C. The resulting solutions were diluted in HNO_3_ (2%, 2 ppb In, 1:300 v/v for brain).

### Imaging and MRT experiments

All operative procedures related to animal care strictly conformed to the Guidelines of the French Government with licenses 380325 and B3818510002 and they were approved by the Ethax committee of the European Synchrotron Radiation Facility (ESRF). All experiments were performed under anesthesia with the following parameters: 3% isoflurane for induction and intraperitoneal injection of xylazine*/*ketamine (64.5*/*5.4 mg kg^−1^) for maintenance.

### Brain tumor inoculation

The 9L gliosarcoma (9LGS) cells were implanted in the brain of male Fisher 344 rats (Charles River, France). Anesthetized animals were placed on a stereotactic frame, and 10^4^ 9LGS cells suspended in 1 μL culture medium with antibiotics were injected through a burr hole in the right caudate nucleus (3.5 mm lateral to the *bregma*, 6 mm below the skull surface)^28^,^31^.

### Preparation of injectable solution

After tangential filtration, a concentrated colloid (GBN in water, [Gd] = 100 mM) was diluted by aqueous solution containing NaCl and hepes in order to obtain an intravenous use solution ([Gd^3+^] = 40 mM, [NaCl] = 145 mM, [hepes] = 10 mM). pH was adjusted to 7.4. Before use, this solution was filtered onto syringe filter with nylon membrane (pore diameter 0.22 μm).

### Drug injection

The aqueous GBN colloid ([Gd^3+^] = 40 mM, [NaCl] = 145 mM, [Hepes] = 10 mM, 1.4 mL) and DOTAREM solution ([Gd^3+^] = 100 mM, V = 0.56 mL) were manually injected in the saphena vein using a syringe and a 26 G needle.

### MR imaging

MRI was performed *using a 7T magnet* (Avance III, Bruker, Germany) at IRMaGe MRI facility (Grenoble, France). Anaesthesia was induced with 3–4% isoflurane and maintained with 1.5–2% isoflurane in a mixture of O_2_/N_2_ (25%/75%).

The imaging protocol consisted of a T_2_-weighted TurboRARE SE sequence (TE = 40 ms, TR = 2500 ms, Field Of View = 3.3 cm, Matrix = 256*256, Slice Thickness = 1 mm), T_1_-weighted FLASH images (TE = 5 ms, TR = 800 ms, Field Of View = 3.3 cm, Matrix = 256*256, Slice Thickness = 1 mm) and dynamic series of transverse slices centered on the rat brain, obtained thanks to a T_1_-weighted MSME sequence (TR/TE = 113.4/10.6 ms, Slice Thickness = 1.5 mm, Field Of View = 5 cm, Matrix = 256*256).

The intravenous injection of GBN ([Gd^3+^] = 40 mM) was performed during the fourth repetition of the dynamic acquisition. The duration of each repetition was about 60 seconds.

### X-ray Fluorescence spectra and maps

Micro-XRF mapping was performed at the X-ray Microscopy beamline ID21 at the European Synchrotron Radiation Facility (Grenoble, France)^32^,^33^. A fluorescence excitation energy of 7.3 keV (above the Gd L-III edge) was selected with a fixed-exit, double-crystal Si(111) monochromator. Kirkpatrick-Baez mirrors were used to focus the beam to a submicron X-ray probe. The beam size was 0.3 × 0.7 μm^2^ with a 9 × 10^10^ photon/s flux. The sample was raster scanned in the micro-beam using a high precision piezoelectric stage to generate a two-dimensional mapping. The X-ray fluorescence photons emitted by the sample were collected using a 10 mm^2^ Silicon Drift Diode (SDD) detector (Bruker, Germany). The microscope was operated under vacuum to avoid air absorption and scattering of the beam.

Regions of interest of 50 × 50 μm^2^ were acquired inside the tumor and in the contralateral region, with 0.5 μm step size. A complete fluorescence spectrum was collected for each pixel, allowing off-line fitting of the spectra and deconvolution of the elemental distributions using the PyMca software^34^.

### Radiation source and MRT setup

MRT was performed at the ID17 biomedical beamline at the European Synchrotron Radiation Facility (Grenoble, France). MRT uses X-rays emitted tangentially to the ring from relativistic electron bunches circulating in a storage ring. The wiggler source produces a white spectrum of photons which extends after filtration (Be (0.5 mm), C (1.5 mm), Al (1.5 mm) and Cu (1.0 mm)) from 50 to 350 keV (mean energy of 90 keV). The quasi-laminar beam is spatially fractionated into an array of microbeams by using an adjustable multislit collimator positioned 41.7 m from the photon source and 100 cm upstream from the head of the animals. Upstream from the multislit collimator, the dose rate within a homogenous field of 10 mm × 10 mm was approximately 90 Gy s^−1^ mA^−1^. Downstream the multislit collimator, the peak entrance dose within the microbeam was ~72 Gy s^−1^ mA^−1^.

### *In vivo* irradiation methods

Ten days after tumor inoculation, the animals were positioned prone on a Kappa-type goniometer (Huber, Germany) in front of the X-rays source, on a home-made Plexiglas frame. Rats were first placed perpendicularly to the beam and received a lateral irradiation, from their anatomically right side to their left. Then, a 90° angle was applied to the motorized goniometer and the second irradiation was performed in the anatomically antero-posterior direction. The field of irradiation was fixed at 10.5 mm height and 8 mm wide and was centered at the theoretical center of the tumor (*i.e.* 3.5 mm lateral to the *bregma*, 6 mm deep from the skull in the right hemisphere). The microbeams were 50 micrometers wide with an on-center distance fixed at 211 microns. The skin entrance dose was set at 400 Gy for the peak and 20 Gy for the valley. The total irradiation procedure lasted about 2 min. Animal immobility during exposure was checked on three control video screens located in the control hutch. A series of 26 rats was divided into untreated rats (n = 5), MRT treated rats without GBN injection (n = 7), treated rats with a 1 h delay between GBN injection and MRT (n = 8), treated rats with a 24 h delay between GBN injection and MRT (n = 6). These delays between drug injection and MRT were chosen according results obtained by MRI. The spatial configuration of the microbeams was checked by radiochromic films (Gafchromic, HD-810).

### Survival analysis

The rats were followed up at the animal facility after the irradiation. At a later tumor stage, rats were euthanized by intra-cardiac injection of pentobarbital sodium less than 1 day before their anticipated death as judged by clinical signs. Some of them were found dead. The time between implantation and death was recorded as survival time (one day was added for euthanized rats). The median survival time (MeST) post-implantation was calculated and Kaplan Meier survival data were plotted versus time after tumor implantation. The increase in lifespan in percent (ILS) characterizes the difference between median survival time for treated and untreated rats divided by the median survival time for untreated rats.

## Additional Information

**How to cite this article**: Dufort, S. *et al.* The High Radiosensitizing Efficiency of a Trace of Gadolinium-Based Nanoparticles in Tumors. *Sci. Rep.* **6**, 29678; doi: 10.1038/srep29678 (2016).
